# Effects of Low-Allergen Wheat and Bayberry Leaf on Wheat Bread: A Comparison with Commercial Wheat

**DOI:** 10.3390/foods14030364

**Published:** 2025-01-23

**Authors:** Yoko Tsurunaga, Eishin Morita

**Affiliations:** 1 Faculty of Human Science, Shimane University, Matsue 690-8504, Japan; 2 Department of Dermatology, Faculty of Medicine, Shimane University, Izumo 693-8501, Japan; emorita@med.shimane-u.ac.jp

**Keywords:** bayberry leaf, proanthocyanidins, allergen protein content, specific volume, antioxidative properties, texture

## Abstract

Gliadin and glutenin wheat proteins are major food allergens. The allergenicity of various wheat products, such as bread, can be reduced by substituting flour with plant-derived tannins. Here, we investigated a technique for reducing the allergenicity of wheat by utilizing the properties of proanthocyanidins (PAs), which strongly bind to proteins. We compared commercial bread wheat (BW), low-allergen wheat (1BS-18 “Minamino Kaori”; 1BS-18M), and bayberry leaves (BBLs). Allergenicity was investigated through enzyme-linked immunosorbent assays (ELISAs) and Western blotting (WB). The immunoreactivity of wheat allergens in both BW and 1BS-18M decreased in a concentration-dependent manner with BBL substitution, and the effect was greatest at 10%. The antioxidative properties also increased with BBL substitution, and the highest antioxidative property was observed at 10%. The specific volumes of both BW and 1BS-18M decreased while the a* value (green to red) increased with increasing BBL substitution. In contrast, no significant differences were observed in the texture of breads with 0% (control), 3%, or 5% BBL substitution. However, 10% BBL substitution led to a significant (*p* < 0.05) reduction in the texture of the bread. Therefore, 5% BBL substitution is optimal for achieving low allergenicity and improved antioxidative properties while maintaining quality.

## 1. Introduction

The most common cause of food allergies is protein. Wheat has mainly two types of proteins: gliadin and glutenin. These two proteins together form gluten, which has a significant impact on the quality of bread and noodles made from wheat [1]. Gliadin is the most potent allergen among wheat proteins and can induce wheat-dependent exercise-induced anaphylaxis (WDEIA), a rare but potentially life-threatening food allergy [2]. Among the various gliadin proteins, ω5-gliadin is the main allergen responsible for WDEIA [2]. Kohno et al. [3] showed by protein analysis that some Chinese Spring deletion lines with a deletion in the short arm of chromosome 1B lack the *Gli-B1* gene, which encodes ω5-gliadin. Based on this observation, Kohno et al. [3] selected a deletion line, 1BS-18, as a suitable candidate for breeding hypoallergenic wheat and showed that its allergenicity was lower than that of commercial wheat flour using the guinea pig wheat challenge model. Using a rat model, Yamada et al. [4] confirmed that the gluten of the low-allergenic wheat line 1BS-18 “Hokushin” (1BS-18H), produced by repeatedly backcrossing 1BS-18 with the widely cultivated wheat variety “Hokushin”, has low allergenicity. In the past, various attempts were made to establish wheat lines lacking allergen-encoding genes, to develop low-allergenic wheat through enzymatic degradation and ion-exchange deamidation, and to produce low-allergenic wheat through thioredoxin treatment. Products obtained using these wheat flour lines can remarkably reduce the reactivity of serum IgE in patients with WDEIA. In some patient groups, however, wheat products obtained using the aforementioned methods were found to be ineffective, and IgE reactivity, though at low levels, was still observed [5].

It is pivotal for patients with food allergies to eliminate allergy-triggering foods for long periods in order to lead a safe daily life. However, doing so can be quite cumbersome and a considerable burden, mentally as well as physically, for both the patient and their caregivers. It also severely impairs the quality of life of the patient and those around them [6]. In previous research, we had attempted to reduce the amount of gliadin in cookies by using plant-origin tannins that can strongly bind to proteins. We showed that it is possible to reduce the amount of wheat allergens in cookies by replacing 3%, 5%, or 10% of the wheat flour with the inner skin of chestnut (CIS) or young persimmon fruit (YPF) [7]. Furthermore, it has also been reported that CIS is effective in reducing the allergenicity of bread [8]. Both CIS and YPF contain a large amount of tannin. However, a major disadvantage of both CIS and YPF is that they are available for only a very short period of time during the year. Chestnuts are harvested in September and October. Hence, CIS can be collected only during this period. Similarly, YPF is collected only in June and July, when the persimmon fruit is in season. To circumvent this issue, we hypothesized that wheat allergens can be reduced if we could use readily available materials other than CIS and YPF that could be harvested all year round. In addition, the usability of these materials can be further increased if we could apply them to foods other than cookies as well. This study is an attempt to examine whether allergens can be reduced using materials that are readily available all year round. This study focuses on bayberry (*Morella rubra* Sieb. et Zucc.) leaves, a material that is discarded in large quantities in Japan. Bayberry is a significant subtropical fruit tree rich in antioxidants [9]. In Japan, bayberry is generally planted as a roadside tree to improve the landscape, and their steamed and dried leaves are sold as a health tea. However, because it grows vigorously, it is regularly pruned. As a result, a large amount of BBLs are discarded. The BBL is mainly composed of PAs, myricitrin, and quercetin-3-rhamnoside [10]. PAs are a type of tannin that strongly binds to proteins. We propose that PAs may have the same effect as that exhibited by CIS and YPF. In addition, because the BBL has very strong antioxidative properties [11], they may transfer this property to the bread as well. Antioxidants have been reported to have the potential to be a solution for controlling chronic diseases such as cardiovascular complications, hypertension, diabetes, and various cancers, so it can be said that consuming foods that are high in antioxidants is extremely important for maintaining good health [12,13,14,15]. However, care should be taken to avoid contamination with pollen during the collection of BBLs. Bayberry pollen is an allergen that has been shown to cause allergic reactions, particularly affecting the nasal and bronchial systems [16]. Therefore, the pollen season should be avoided when collecting BBLs. Furthermore, to investigate the effects of using BBL-containing low-allergen wheat, we compared its effects with those of commercially available wheat for bread-making and low-allergen wheat developed through breeding (1BS-18M).

However, BBL substitution may reduce the quality of bread. The formation of a gluten network is essential for maintaining bread quality. When a part of the flour is replaced with BBL, the BBL components, such as PAs and monomeric polyphenol, may change the gluten network. For example, PA forms cross-links with wheat gluten, thereby increasing its polymer size and strength. In addition, PA has a higher binding affinity to glutenin than to gliadin, particularly to high-molecular-weight glutenin subunits rather than low-molecular-weight glutenin subunits, and to ω-gliadin rather than α- and γ-gliadins [17]. Several studies have also claimed that oligomeric procyanidin (a type of PA) improves the physicochemical properties of flour, resulting in a more compact and denser gluten microstructure, which changes the rheological properties, molecular weight distribution, secondary structure, and thermal stability of gluten [18]. Hence, we can suggest that the PAs in the BBLs may either stabilize or destabilize the gluten network. Therefore, in this study, we investigate the extent to which the BBL-substitution rate contributes to allergen reduction and the antioxidative properties of bread, as well as its impact on the appearance, color, specific volume, and texture of bread.

## 2. Materials and Methods

### 2.1. Preparation of BBL Powder

BBLs harvested at Shimane University (Matsue City, Japan) in June 2022 were used. The BBL samples were freeze-dried and then made into a powder using an Oster blender (Osaka Chemical Co., Ltd., Osaka, Japan). The BBL powder was sieved through a 1 mm mesh and sealed in an aluminum laminate plastic bag (Lami Zip AL-16; Hoshizai Nihon Co., Ltd., Tokyo, Japan).

### 2.2. Wheat Flour Used in Bread Production

We used two varieties of wheat for producing bread: commercially available BW wheat and 1BS-18M, a low-allergen wheat. BW flour refers to the flour from which the bran has been removed, while 1BS-18M flour refers to whole-wheat flour. Ideally, in order to match the conditions, 1BS-18M should not be whole-wheat flour, but rather flour from which the bran has been removed. However, because 1BS-18M is a low-allergen wheat, a special flour mill is needed to prevent the contamination of allergens from other flours. Currently, it is difficult to prepare equipment dedicated to 1BS-18M that can completely remove bran from 1BS-18M. Therefore, whole-wheat flour was used.

### 2.3. Characteristics of BBL and Wheat Flour

Scanning electron microscopy (SEM) microscopy was used to observe the surface structure of the sample powder. The sample was fixed to an SEM specimen stand (Type-HM; Nissin EM Corporation, Tokyo, Japan) with double-sided carbon tape for SEM (8 mm × 20 m; Nissin EM Corporation, Tokyo, Japan). Gold deposition was carried out, and the specimens were observed via SEM (JSM-IT800SHL; JEOL Ltd., Tokyo, Japan) at an acceleration voltage of 10 kV and a magnification of 800×. The particle size distribution of the samples was measured using a laser-scattering particle size analyzer (Partica LA-960V2; Horiba, Kyoto, Japan). Ethanol (99.5%) (Nacalai Tesque Corporation, Kyoto, Japan) was used as the dispersant. Samples were added directly to ethanol without suspension, and no ultrasonic treatment was performed. Median and average particle diameters were measured from the particle size distribution of the samples. The color of the samples was measured using a spectrum colorimeter (CR-13; Konica-Minolta Co., Tokyo, Japan). We commissioned an external company to analyze the general composition and dietary fiber content of the raw materials BBL, BW, and 1BS-18M. Both BBL and BW were analyzed by the Bureau Veritas FEAC (Izumo City, Japan), while 1BS-18M was analyzed by the Japan Food Analysis Center (Tokyo, Japan). The following measurement methods were used for each component: for protein, the Kjeldahl method (protein conversion factor: 6.25); for lipid, the acid decomposition method; for carbohydrate, the subtraction method (100 g − (protein + fat + moisture + ash)); for dietary fiber, the enzyme and gravimetric method; for ash, the direct dry-heating method; and for moisture, the normal-pressure heating and drying method. The energy was calculated by using the modified Atwater method, and the following values were obtained: protein, 4 kcal/g; fat, 9 kcal/g; and carbohydrate, 4 kcal/g.

### 2.4. Bread Production Methods

To clarify the effects of BBL substitution on the allergenicity and quality of bread, bread production tests were conducted using a kneader (PK-601; Nihon Kneader Co., Fujisawa City, Japan) that allows the time and temperature to be set for each production process. The dough was kneaded twice using the sponge–dough method. First, the following ingredients were added to the kneader: 120 g flour, 6 g dry yeast, 20 g sugar, and 204 g water. After kneading for 5 min, the dough was allowed to rest for 10 min at 25 °C. Then, 180 g of flour and 4 g of salt were incorporated and kneaded for 5 min. Next, 15 g of butter was incorporated into the dough and kneaded for another 10 min at 25 °C. The dough was subjected to a primary rise step for 30 min at 35 °C using the steam function of a convection heat oven (Convection Oven NE-CBS2700; Panasonic Corporation, Osaka, Japan). Next, after punching down the dough in a kneader for 20 s, the dough was divided into two pieces and left to undergo a secondary fermentation step for 15 min at 35 °C. The dough was lightly deflated to release the gas, placed in a 1-loaf mold (9.7 (D); 19.8 (W); 9.6 (H) cm), and fermented for 40 min at 35 °C. It was then baked at 160 °C for 10 min, 190 °C for 20 min, and 200 °C for 5 min. BBL was added to the wheat flour in two portions. In the first portion, 3%, 5%, and 10% of the 120 g of flour was replaced with BBL. In the second portion, 3%, 5%, and 10% of the 180 g of flour was replaced with BBL. After baking, the dough was taken from the mold, transferred to a polyethylene bag (thickness 0.04 mm), and cooled for 24 h at 25 °C with the top open. Powdered samples of bread dried for 72 h at 25 °C were used for evaluating the allergens. In addition, the total polyphenol content (TPC) and the activity of two antioxidants from both bread types were determined.

### 2.5. Evaluation of the Immunoreactivity of Wheat Proteins

#### 2.5.1. ELISA

Two types of ELISA kits were used to measure the wheat proteins: FASPEK ELISA II^®^ for gliadin and FASTKIT ELISA Ver. III^®^ for wheat. The former is an ELISA kit developed by Morinaga Bio-Science Laboratory (Yokohama City, Japan) and the latter by NH Food (Tsukuba City, Japan). Both of these ELISA kits were reported as analytical tools in Japan in 2002 [19]. FASPEK KIT II^®^ uses polyclonal antibodies, and gliadin is the target protein in wheat [19]. FASTKIT ELISA Ver. III^®^ uses polyclonal antibodies against multiple wheat protein components to detect all the protein allergens [19]. Both tests were carried out in accordance with the manufacturer’s instructions (Morinaga Institute of Life Sciences, 2017; Center, and Ver ELISA, 2014) [20,21] attached to each kit. After extraction by horizontal shaking at 20 °C for 20 h (MMS-1020; EYELA, Tokyo, Japan), the pH was adjusted, and the sample was centrifuged at 3000× *g* for 20 min (LCX-100; Tomy Seiko Co., Ltd., Nerima-ku, Japan). The supernatant was diluted as necessary and used for analysis. For FASPEK KIT II^®^, diluted standard and sample solutions were added to a 96-well microplate coated with antibodies and incubated at room temperature (20 °C) for 1 h. After washing with a microplate washer (Cat # 5165000; Thermo Fisher Scientific, Vantaa, Finland), the peroxidase-labeled antibody solution was added and left to stand at 20 °C for 30 min. After a second wash, 3,3,5,5-tetramethylbenzidine solution was added to the aforementioned solution and left to stand for 20 min at 25 °C. Then, 1N sulfuric acid was added to stop the reaction. The microplate reader was used to measure the absorbance at 450 nm (SH-9000Lab; Corona Electric, Hitachinaka City, Japan) using 630 nm as the reference wavelength. In the case of FASTKIT ELISA Ver. III^®^, the diluted standard solution and the sample solution were added to a 96-well microplate coated with antibodies and incubated at room temperature (20 °C) for 1 h. The plate was washed again with a washing solution, the streptavidin–peroxidase reagent was added, and the resulting solution was incubated at 20 °C for 30 min. The plate was washed again, the chromogenic enzyme substrate was added, and the resulting solution was incubated at 20 °C for 20 min. The reaction was stopped using 0.5 N sulfuric acid, and the absorbance at 450 nm was measured with a microplate reader using 630 nm as the reference wavelength. Extraction was performed twice per treatment. Measurements were taken twice per extraction. The results were expressed as the mean ± standard error (SE) per dry weight (DW).

#### 2.5.2. WB Analysis

Western blotting was performed using the method described previously [2]. ω5-gliadin was purified from the water-insoluble fraction of commercial wheat flour using the method described previously [2]. The samples were dissolved in a sample buffer and heated at 95 °C for 5 min. To determine the protein content in the samples, the proteins were extracted using the RC DC Protein Assay Kit (Bio-Rad Laboratories, Hercules, CA, USA) to remove β-mercaptoethanol. The protein concentration was determined following the Lowry method using the DC Protein Assay Kit (Bio-Rad Laboratories)

The sample dissolved in the sample buffer was separated through a 12.5% acrylamide gel using sodium dodecyl sulfate–polyacrylamide gel electrophoresis (SDS-PAGE). The proteins were visualized through Coomassie Brilliant Blue (CBB) staining. For immunoblotting, the proteins were electrophoretically transferred to a polyvinylidene fluoride (PVDF) membrane (Immobilon-*p*; Merck Millipore, Burlington, MA, USA) and reacted with polyclonal rabbit anti-ω5-gliadin IgG antibodies. A specific antibody against ω5-gliadin was produced as a rabbit polyclonal antibody using a peptide (KQQSPEQQQFPQQQIPQQQ) containing the three IgE-binding epitopes of ω5-gliadin as an antigen [3]. After reacting with horseradish peroxidase-conjugated donkey anti-rabbit IgG (GE Healthcare, Buckinghamshire, UK), the ω5-gliadin bound to anti-ω5-gliadin IgG was visualized using ECL Prime Western Blotting Detection Reagents (Amersham, Buckinghamshire, UK).

### 2.6. TPC and Antioxidative Activity Assays

The antioxidative activity of the bread was assessed using two methods: 2,2-diphenyl-1-picrylhydrazyl (DPPH) and hydrophilic oxygen-radical absorbance capacity (H-ORAC) assays. Folin–Ciocalteu reagent solution (2 N), DPPH (95%), Trolox (97%), 2,2′-azobis(2-amidinopropane) dihydrochloride (AAPH, 95%), and ethanol solution (99.5%) were purchased from Wako Chemicals Ltd. (Osaka, Japan). Sodium fluorescein salt (1 mg/mL in pure water) was purchased from Sigma-Aldrich (St. Louis, MO, USA). Catechin (CTN) (≥98%, powder) was purchased from Funakoshi Co. (Tokyo, Japan). The TPC, DPPH, and H-ORAC values of the breads were measured. The samples were extracted in 60% ethanol for 2 h at 40 °C with shaking, in accordance with a previously reported protocol [22]. The TPC was measured using the Folin–Ciocalteu method [23]. The TPC was expressed as mg CTN equivalent/100 g dry weight (mg CTN eq/100 g DW). To determine the PA content, 30 mg of polyvinylpyrrolidone (PPVP) per milliliter of the extract solution was added to adsorb PAs. This value was then subtracted from the TPC [24]. The antioxidative activity of the extracts was analyzed using the DPPH assay [25,26] and H-ORAC assay [27]. The DPPH and H-ORAC values were expressed as µmol Trolox equivalents/g of DW (µmol TE/g DW). The extraction was performed twice per treatment, and the measurements were performed in triplicate per extraction. The results were expressed as the mean ± SE.

### 2.7. HPLC and UHPLC-ESI-MS/MS Analyses

The 60% ethanol extract of BBL was analyzed by a quantitative HPLC system (LaChrom; Hitachi Ltd., Chiyoda-ku, Japan) by comparison with standard compounds using an InertSustainSwift C18 column (4.6 × 150 mm) (GL Sciences Inc., Tokyo, Japan). The analysis conditions were as follows: UV detection, 280 nm (0–7.5 min) and 370 nm (7.5–60 min); column oven temperature, 40 °C; flow rate, 1.0 mL/min; mobile phases, (A) 0.1% formic acid/water and (B) 0.1% formic acid/acetonitrile; gradient condition, 0 min → (100:0) → 2 min (90:10) → 15 min (65:35) → 20 min (65:35) → 20.10 min (5:95).

LC-MS/MS analysis was also performed to determine the substances corresponding to the major peaks obtained by HPLC. A tandem mass spectrometer microTOF-QII equipped with an electrospray ion source (Bruker Daltonik, Bremen, Germany) coupled with an ultra-high-performance liquid chromatograph (Nexera, Shimadzu, Nakagyo-ku, Japan) was used. The mobile phase A was 0.1% (*v*/*v*) formic acid, and mobile phase B was 0.1% (*v*/*v*) formic acid in 99.8% acetonitrile. Samples were eluted onto a C18 HPLC column (InertSustainSwift C18 column HP 3 µm, 2.1 mm × 150 mm; GL Sciences, Japan). The gradient was as follows: 0 min, 10% B; 2 min, 10% B; 15 min, 40% B; 20 min, 40% B; 20.1 min, 95% B; 25 min, 95% B; 25.1 min, 15% B; 35 min, 15% B. The MS analysis was carried out in a data-dependent acquisition mode, using the top three precursors and the CID fragmentation technique for identification. The LC-MS system was controlled by the OtofControl v3.2 and HyStar v3.2 (Bruker Daltonik, Bremen, Germany) software. The MS analyzer settings included the source (positive mode; capillary voltage: 4500 V; dry gas: 8.0 L/min, dry temperature: 180 °C) and tune page (funnel 1 RF: 150 Vpp; funnel 2 RF: 200 Vpp; in-source CID energy: 0 V; hexapole RF: 200 Vpp; quadrupole ion energy: 5 eV; collision energy: 10 eV; collision RF: 500 Vpp; transfer time: 98.4 µs; pre-pulse storage: 1 µs; spectra rate: 1 Hz). The MS/MS settings were the following: auto MSMS, on; three precursor ions; the threshold for switching from MS to MSMS mode, 5000 cts; active exclusion after two spectra; release after 1 min; excluded mass range of precursors, 50–200 *m*/*z* and 800–1500 *m*/*z*. Data were collected over the mass range of 50–1500 *m*/*z* with an acquisition rate of 1 Hz for survey MS and in a range of 200–800 *m*/*z* for MS/MS, depending on the precursor intensity.

### 2.8. Bread Quality Assessment

#### 2.8.1. Appearance and Color

The bread samples were sliced into 2 cm thick pieces. To observe their appearance, the cut sample surfaces were documented using a digital camera (WG-40 W; Ricoh Company, Ltd., Tokyo, Japan). To examine the internal structure, stereomicroscopic images (DS-L3; Nikon Corporation, Tokyo, Japan) were also used. The color in the cut surface of the bread was measured using a spectrum colorimeter (CR-13; Konica-Minolta Co., Tokyo, Japan). The three measurement items are L* (black to white), a* (red to green), and b* (yellow to blue). Ten measurements were performed per treatment. The results were expressed as the mean ± SE.

#### 2.8.2. Specific Volume

The volume of the sample loaves of bread was determined using the rapeseed displacement method (American Association of Cereal Chemists International method 10-05.01) [28]. To calculate the specific volume, the volume of the bread was divided by the weight of the bread. The specific volume was measured three times per treatment and expressed as the mean ± SE.

#### 2.8.3. Textural Properties

Textural properties were measured using a creep meter (RE2-33005B; Yamaden Co., Ltd., Tokyo, Japan). Samples for measuring the textural properties were cut into 2 × 2 × 2 cm pieces from the center of the bread using an ultrasonic cutter (SUW-30CTL; Suzuki Manufacturing Co, Shizuoka, Japan). Texture measurements were performed on a creep meter equipped with a 20 N load cell and a 30 mm diameter acrylic resin plunger at 10 mm/s velocity, 50% strain rate, and 20 ± 1 °C. The maximum load, cohesiveness, and gumminess of each sample were quantified using texture analysis software (Ver. 2.2; Yamaden Co., Ltd., Tokyo, Japan). Ten measurements were taken per treatment. The results were expressed as the mean ± SE.

### 2.9. Statistical Analysis

Each data was statistically analyzed using SPSS version 28.0 (SPSS Inc., Chicago, IL, USA). To identify differences among the groups, one-way analysis of variance (ANOVA) was used, followed by Tukey’s test for multiple comparisons. A value of *p* < 0.05 was considered statistically significant.

## 3. Results and Discussion

### 3.1. Characteristics of BBL and Wheat Flour

**Figure 1:**
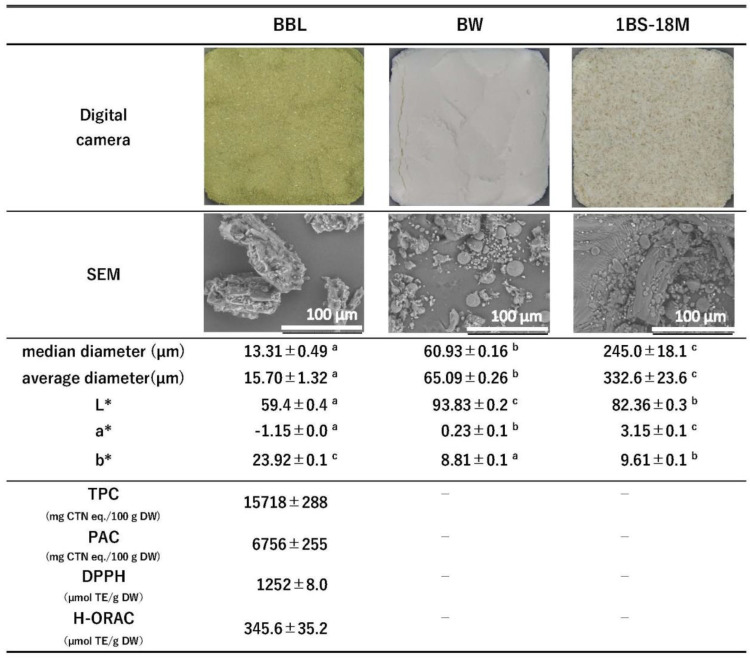
Digital camera images, scanning electron microscopy (SEM) images, particle size, and color differences in the experimental material. BBL, bayberry leaf; BW, bread wheat; 1BS-18M, 1BS-18 “Minamino Kaori”; TPC, total polyphenol content; PAC, proanthocyanidin content; DPPH, 2,2-diphenyl-1-pycrylhydrazyl; DPPH value, DPPH radical-scavenging activity value; H-ORAC value, hydrophilic oxygen oxygen-radical absorbance capacity value. Values represent the mean ± standard error (Median diameter and Average diameter, *n* = 3; Color, *n* = 10; TPC, PAC and DPPH, *n* = 6; H-ORAC, *n* = 4). The different letters indicate statistical differences (*p* < 0.05).

**Figure 2:**
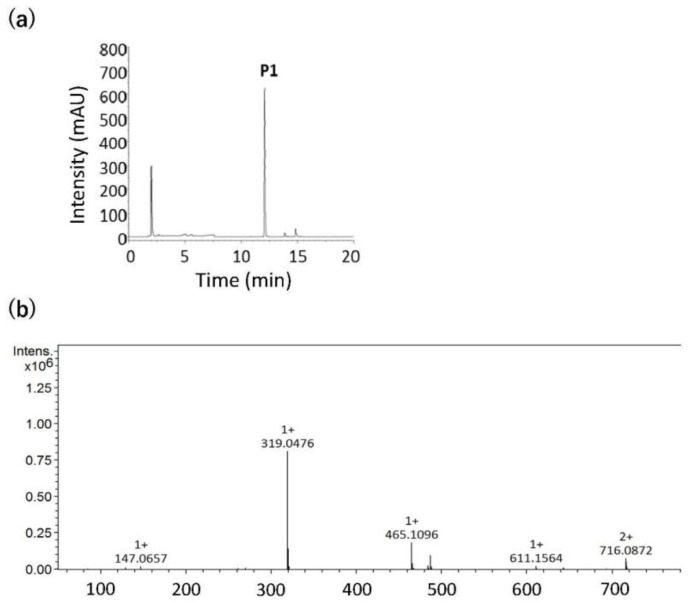
(**a**) High-performance liquid chromatography (HPLC) chromatogram of the 60% ethanol extract of bayberry leaves (BBL), and (**b**) the mass spectrum of the peak (P1 of Figure 2a).

The properties of the raw materials used in this experiment, i.e., those of BBL, BW, and 1BS-18M, are summarized in Figure 1. BBL had a very high TPC (15718 ± 288 mg CTN eq./100 g DW). PVPP was used to measure the total amount of PA, and the PA content was 6756 ± 255 mg CTN eq./100 g DW. A difference of 8962 CTN eq./100 g DW between the TPC and the PA content suggests the involvement of polyphenolic compounds other than PAs. Therefore, the polyphenol components of BBL were measured using HPLC, and the chromatogram is shown in Figure 2a. Based on the LC/MS/MS results (Figure 2b) and previous reports [29], we determined that the single largest peak of Figure 2a (P1) belongs to myricetin-3-O-rhamnoside (myricitrin). The *m*/*z* 465.1096 observed in the mass spectrum of Figure 2b was myricetin-3-O-rhamnoside (MW = 464 Da), and it is thought that the myricetin aglycone *m*/*z* 319.0476 (MW = 318 Da) was observed after the rhamnoside residue (146 Da) was cleaved. Our results showed that BBL contained 2326 ± 19.3 g/100 g DW of myricitrin. Other than myricitrin, we also found quercetin-3-O-galactoside, quercetin-3-O-rhamnoside, and an unknown component [11], although in lower amounts than that of myricitrin. Thus, the TPC and the large difference between the STC and TPC values were presumably due to the effect of low-molecular-weight polyphenols. In addition, BBL had very high DPPH and H-ORAC values. (1252 ± 8.0 and 345.6 ± 35.2 μmol TE/g DW, respectively).

A previous study reported that BBL exhibits very strong antioxidative properties [10,29], which is consistent with the results of our study. We evaluated two types of wheat: commercial BW and hypoallergenic 1BS-18M wheat. Figure 1 shows the SEM image of the raw materials used in the present study. The digital image shows BBL as a fine green powder, whereas the SEM image shows that BBL comprises angular and irregular particles, with a particle size of 100–150 µm. Round objects of 20 µm were observed in commercial BW. Based on previous reports, we can infer that these round objects were wheat starch grains [30]. In the case of the wholemeal flour 1BS-18M, the presence of bran could be confirmed with the naked eye. In addition, SEM observation revealed that the bran particles were over 100 µm in size (Figure 1). When the particle sizes of each were measured, the median diameter and average diameter were 1BS-18M > BW > BBL. Large fragments were observed in the BBL in the SEM images, but detailed examination of the particle size distribution revealed a mixture of fragments of various sizes, including many fragments of about 5-20 µm, which is thought to be why both the median and average diameters were low (Figure 1). A difference in coloration was also observed, with BW having a remarkably high L* value, indicating lightness, and a bright white color. In contrast, 1BS-18M had a high b* value and a yellowish color, presumably due to its high bran content (Figure 1). Table 1 shows the general composition and the amount of dietary fiber in the raw materials. As BBL is a dried plant leaf, it has a significantly high dietary fiber content (53.2 g/100 g). The protein contents of BW and 1BS-18M were comparable (13.1 and 12.8 g/100 g, respectively). However, the lipid, dietary fiber, and ash contents of 1BS-18M were higher than those of BW. Previous research on the topic has generated a large amount of data that suggest that whole-wheat flour has a higher amount of total dietary fiber [31,32,33,34], ash [31,32,33,34], and lipid [33] than those present in refined wheat. Our study arrives at similar results. We presumed that these results were obtained because whole-wheat flour contains a large amount of bran, which is rich in dietary fiber and minerals, and germ, which is rich in fats and oils.

### 3.2. Evaluation of the Immunoreactivity of Wheat Proteins

#### 3.2.1. WB Analysis

**Figure 3:**
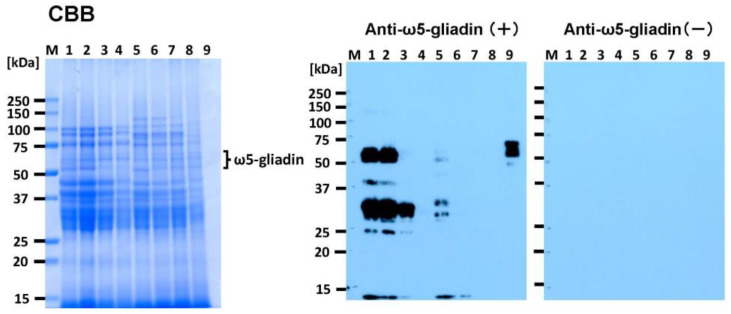
Detection of immunoreactivity in BBL bread using Western blotting with polyclonal rabbit anti-ω5-gliadin IgG antibodies. Bread samples (10 µg/lane) and purified ω5-gliadin (0.25 µg/lane) were electrophoresed, transferred, and blotted using anti-ω5-gliadin IgG antibodies. Lane M, molecular weight marker; lane 1, BW bread without tannin material (Control); lane 2, 3% BBL BW bread; lane 3, 5% BBL BW bread; lane 4, 10% BBL BW bread; lane 5, 1BS-18M bread without tannin material; lane 6, 3% BBL 1BS-18M bread; lane 7; 5% BBL 1BS-18M bread; lane 8, 10% BBL 1BS-18M bread; lane 9, purified ω5-gliadin. BBL, bayberry leaf; BW, bread wheat.

CBB staining of SDS-PAGE gels showed that all the bread samples (lanes 2–8) produced several protein bands in the molecular weight range of 25–100 kDa that were similar to the protein patterns observed for bread without BBL substitution (lane 1). The intensity of the bands decreased with increasing substitution of BBL (lanes 1–4 for BW bread and lanes 5–8 for 1BS-18M bread in Figure 3). This indicates that the binding of PAs to wheat proteins inhibits CBB staining. When blotted with anti-ω5-gliadin IgG antibodies, the immunoreactivity of gluten proteins in the BW bread decreased with increasing BBL substitution (lanes 1–4 in Figure 3). No immunoreactivity was observed in BW bread at 10% BBL substitution (lanes 4 in Figure 3). In the 1BS-18M bread, no immunoreactivity was observed in any of the treatments with BBL substitution (lanes 6–8 in Figure 3).

#### 3.2.2. ELISA

**Figure 4:**
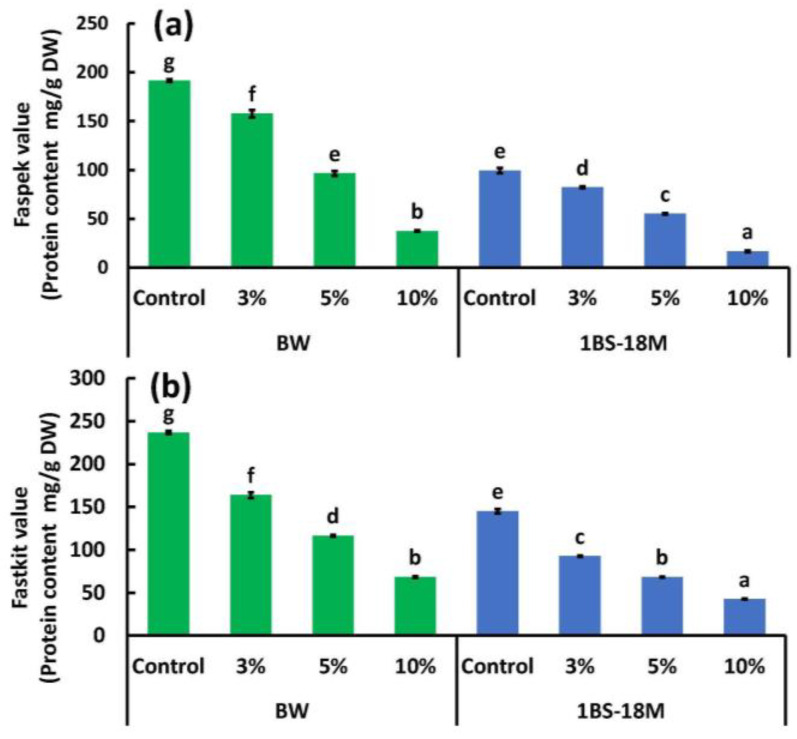
Effect of BBL substitution on (**a**) FASPEK values, and (**b**) FASTKIT values. FASPEK value, value obtained using the FASPEK ELISA II^®^ for gliadin kit; FASTKIT value, value obtained using the FASTKIT ELISA Ver. III^®^ kit for wheat; BW, bread wheat; 1BS-18M, 1BS-18 “Minaminokaori”; BBL, bayberry leaf. The values are expressed as the standard reagent equivalents per dry weight. The results were analyzed using ANOVA followed by Tukey’s test for multiple comparisons. Different letters indicate significant differences at *p* < 0.05. Data are expressed as the mean ± SE (*n* = 4).

The effect of BBL substitution on the total content of allergen proteins in the bread samples was evaluated using two ELISA kits: FASPEK ELISA II^®^ and FASTKIT III^®^ (Figure 4). In Japan, these two ELISA kits are employed as official allergen screening evaluation methods because of their high stability for five major allergenic components, based on the results of cross-analysis conducted by 10 research institutes [35]. FASPEK KIT II^®^ uses a polyclonal antibody to detect specific purified proteins or individual proteins of specific components (hereafter referred to as FASPEK values). In wheat, gliadin is targeted [19]. FASTKIT ELISA Ver. III^®^ uses polyclonal antibodies against multiple antigens to detect the complete set of allergenic proteins (hereafter FASTKIT values) [19]. FASPEK values for BW ranged from 37.5 (BBL10% replacement) to 191.5 mg/g DW (control), while those for 1BS-18 ranged from 16.7 (BBL10% replacement) to 99.5 mg/g DW (control), indicating that, overall, 1BS-18M had lower FASPEK values than BW (Figure 4a). The FASPEK values for BW are highest in the BBL-free control (191.5 ± 1.4 mg/g DW). As the BBL substitution was increased to 3%, 5%, and 10%, the FASPEK values for BW decreased significantly to approximately 82%, 50%, and 20% of the control, respectively (Figure 4a). The 1BS-18M FASPEK values were highest in the control (99.5 ± 3.2 mg/g DW; 52% of the BW control) and significantly decreased with increasing BBL substitution: 43%, 29%, and 9% of the BW control at 3%, 5%, and 10% of BBL substitution, respectively (Figure 4a). The FASTKIT values measured using FASTKIT ELISA Ver. III^®^ are shown in Figure 4b. The FASTKIT values for both BW and 1BS-18M significantly decreased with increasing BBL substitution (Figure 4b) (*p* < 0.05). The FASTKIT values for BW ranged from 68.4 (10% BBL) to 236.8 mg/g DW (control), while those for 1BS-18M ranged from 42.6 (10% BBL) to 145.1 mg/g DW (control). Notably, 1BS-18M has lower values overall (Figure 4b). The FASTKIT values for BW were highest in the controls (236.8 ± 1.7 mg/g DW) and decreased to approximately 69%, 49%, and 29% of the controls at 3%, 5%, and 10% BBL substitution, respectively (Figure 4b). The FASTKIT values were highest in the control (145.1 ± 2.7 mg/g DW; 61% of the BW control) and decreased with increasing BBL substitution, reaching 39%, 29%, and 18% of the BW control value at 3%, 5%, and 10% BBL substitution, respectively (Figure 4b). BBL contains the flavonoids myricitrin [10] and PAs [29]. PAs have phenolic hydroxyl groups and hydrophobic regions that form complexes with hydrophobic amino acids and the carbonyl groups of the respective protein [17]. In addition, BBL contains a large amount of PAs (a type of tannin) (Figure 1), which may be responsible for reducing the allergenicity of bread. BBL is also rich in myricetin 3-O-α-L-rhamnopyranoside (myricetin; molecular formula C_21_H_20_O_12_). Girard et al. [17] investigated the interaction of PAs (from sorghum and grape seeds) with wheat gluten proteins and reported that monomeric CTN did not precipitate the gluten fraction and that PAs from sorghum formed a strong complex with the protein. From our experiments, it is also unlikely that myricitrin, a monomer, would bind to protein. Therefore, we thought that the binding of PA and allergen protein was involved in the reduction of allergen content. The binding affinity of PAs to proteins is impacted by the size and shape of the protein and has been shown to have a strong affinity for larger proteins with less compact shape [36]. Gliadins consist of α-gliadin (MW ≅ 31 kDa), γ-gliadin (MW ≅ 35 kDa), and ω-gliadin (MW ≅ 44-80 kDa) [37]. Of these, PAs preferentially bind to ω-gliadin [17]. The Western blot evaluation showed that the ω5-gliadin band disappeared, but the ELISA only reduced the FASPEK and FASTKIT values. This is because FASPEK ELISA II^®^ [35], which detects α-gliadin and γ-gliadin, and FASTKIT ELISA VER. III^®^, which detects all the wheat allergen proteins using polyclonal antibodies against multiple antigens other than gliadin, failed to bind the PAs in the BBL. Hence, it can be suggested that allergens that could not bind to PAs in the BBL remained in the bread sample. Similar to the PAs in sorghum [17], those in BBLs may preferentially bind to ω-gliadin and do not bind sufficiently to other proteins. However, this experiment did not reveal which protein species in wheat bind with each other and at what strength. This should be verified in the future. The ELISA results only showed that the FASPEK and FASTKIT values were significantly reduced compared with that of the controls (Figure 4). In contrast, the immunoblotting results showed that BBL substitution almost completely abolished the immunoreactivity (Figure 3). Hence, it can be inferred that BBL substitution is sufficiently effective for patients with WDEIA, in whom ω5-gliadin is the causative agent of the allergy.

### 3.3. TPC, DPPH, and H-ORAC Values

**Figure 5:**
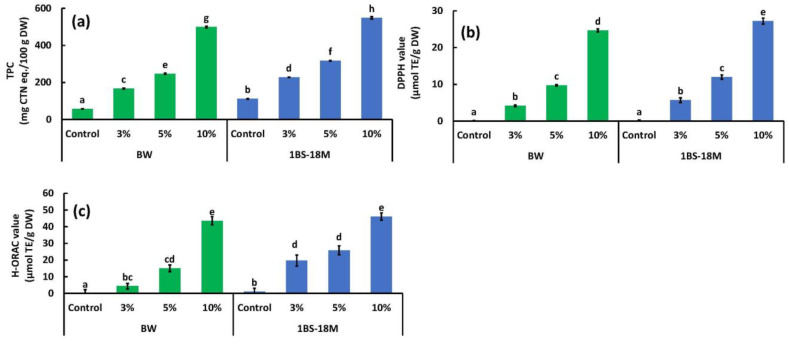
Effects of BBL substitution on (**a**) TPC, (**b**) DPPH, and (**c**) H-ORAC values of bread. TPC, total polyphenol content; DPPH, 2,2-diphenyl-1-pycrylhydrazyl; DPPH value, DPPH radical-scavenging activity value; H-ORAC value, hydrophilic oxygen-radical absorbance capacity value. The values are expressed as the standard reagent equivalents per dry weight. The results were analyzed using ANOVA followed by Tukey’s test for multiple comparisons. Different letters indicate significant differences at *p* < 0.05. Data are expressed as the mean ± SE. TPC and DPPH, *n* = 6; H-ORAC, *n* = 4.

Figure 5 shows the TPC (Figure 5a), DPPH (Figure 5b), and H-ORAC (Figure 5c) values of bread produced using the BW or 1BS-18M wheat type. The TPC of BW bread increased with increasing BBL substitution, with 167, 247, and 499 mg CTN eq./100 g DW at 3%, 5%, and 10% BBL substitution, respectively, compared with 58 mg CTN eq./100 g DW for the control (Figure 5a). Similarly, the TPC of 1BS-18M bread also increased as the BBL-substitution rate increased, with 228, 317, and 549 mg CTN eq./100 g DW at 3%, 5%, and 10% BBL substitution, respectively, compared with 112 mg CTN eq./100 g DW for the control (Figure 5a). The DPPH value of BW bread increased with increasing BBL substitution, with 4, 10, and 25 µmol TE/g DW at 3%, 5%, and 10% BBL substitution, respectively, compared with 0 µmol TE/g DW for the control (Figure 5b). The DPPH value of 1BS-18M bread also increased as the BBL substitution rate increased, with 6, 12, and 27 µmol TE/g DW at 3%, 5%, and 10% BBL substitution, respectively, compared with 0 mg µmol TE/g DW for the control (Figure 5b). The H-ORAC value of BW bread increased with increasing BBL substitution, with 4.3, 15.1, and 43.5 µmol TE/g DW at 3%, 5%, and 10% BBL substitution, respectively, compared with 0 µmol TE/g DW for the control (Figure 5c). The H-ORAC values of 1BS-18M bread of were 19.7, 25.9, and 46.1 µmol TE/g DW at 3%, 5%, and 10% BBL substitution, respectively, compared with 1.1 mg µmol TE/g DW for the control (Figure 5c). Hence, the TPC, DPPH, and H-ORAC values became significantly higher with increasing BBL substitution for all treatments, except for one (the H-ORAC values in 1BS-18M remained the same at 3% and 5% BBL substitution) (*p* < 0.05). The increase in the activity of the TPC, DPPH, and H-ORAC values with increasing BBL substitution can be attributed to the activity of the raw material, BBL. BBL has very high values of TPC, DPPH, and H-ORAC: 15,718 mg CTN eq/100 g DW, 1252 µmol TE/g DW, and 345.6 µmol TE/g DW, respectively (Figure 1).

Antioxidants play an important role in preventing various diseases, such as cardiovascular diseases, cancers, Alzheimer’s disease, and cataracts, by reducing oxidative stress and protecting cells from damage [13,38,39,40]. In addition, they can help slow down the aging process. Consequently, they are attracting a great deal of attention [41]. Myricitrin, present in large amounts in BBL, has strong antioxidative properties [42,43]. A previous study extracted PAs from BBL and administered them to rats and achieved an anti-obesity effect [44]. Furthermore, the flavonoids present in BBL exhibit very strong glucosidase inhibitory activity. Hence, they can be used as an alternative treatment for diabetes [11]. It is highly likely that BBL substitution will confer these functions to bread as well. These results suggest that the addition of BBL can make bread healthier than the non-BBL-containing bread types.

### 3.4. Appearance, Microscopic Images, and Color

**Figure 6:**
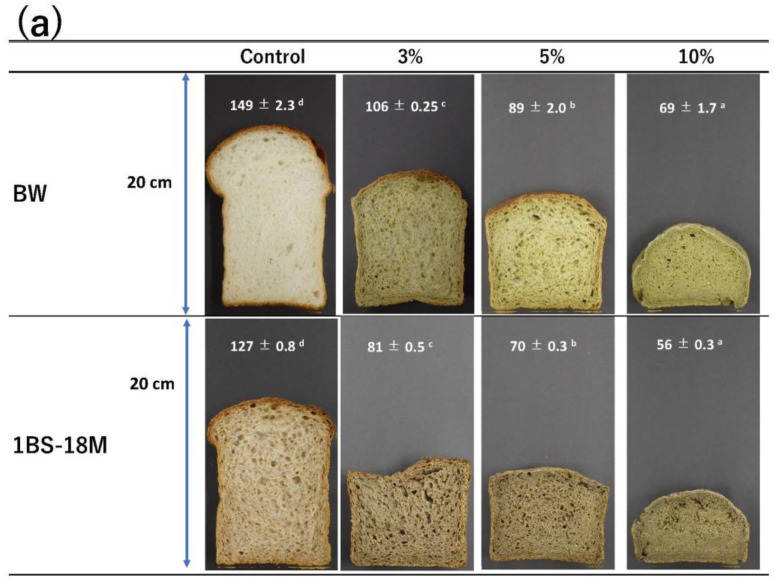

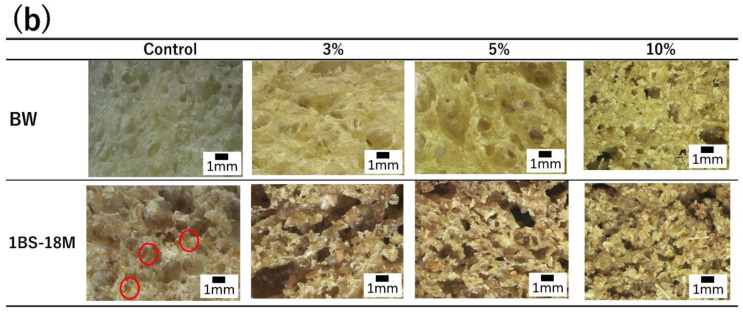
Effect of BBL replacement rate on the (**a**) appearance, height, and (**b**) actual microscopic photographs of bread. BW, bread wheat; 1BS-18M, 1BS-18 “Minaminokaori”; BBL, bayberry leaf. The results were analyzed using ANOVA followed by Tukey’s test for multiple comparisons. Different letters indicate significant differences at *p* < 0.05. Height data are expressed as the mean ± SE (*n* = 4). The red circle indicates bran.

**Figure 7:**
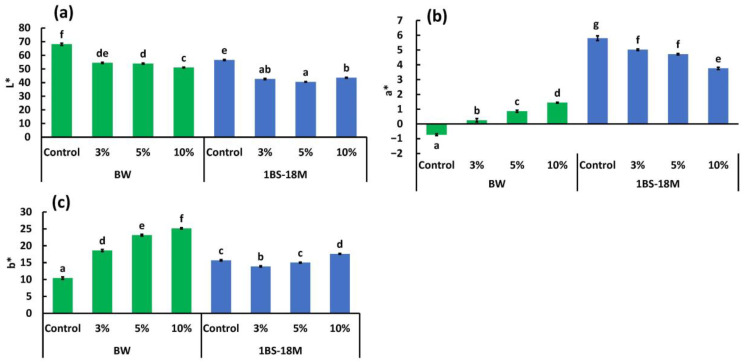
Effect of BBL substitution rate on (**a**) L*, (**b**) a*, and (**c**) b* values. BW, bread wheat; 1BS-18M, 1BS-18 “Minaminokaori”; BBL, bayberry leaf. The results were analyzed using ANOVA followed by Tukey’s test for multiple comparisons. Different letters indicate significant differences at *p* < 0.05. Height data are expressed as the mean ± SE (*n* = 10).

Figure 6 shows the appearance (a) and stereomicrographs (b) of breads produced using BW and 1BS-18M. Figure 7 shows the color results. In the stereomicroscopic images, while the control BW bread exhibited numerous small, raised bubbles measuring 1–2 mm in diameter, the quantity of bubbles diminished as the BBL substitution increased (Figure 6b). In particular, with 10% BBL substitution, the number of bubbles was remarkably low in both the BW and 1BS-18M bread varieties (Figure 6b). In the 1BS-18M bread, many particles with a diameter of 1–2 mm that could be considered bran were observed with the naked eye (red circle in Figure 6b). The height of the BW bread varied from 149 ± 2.3 mm (control) to 69 ± 1.7 mm (BBL 10%), while that of the 1BS-18M bread varied from 127 ± 0.8 mm (control) to 56 ± 0.3 mm (BBL 10%). It is generally known that adding bran reduces the volume of bread [45]. Bran particles destabilize the film that separates air bubbles, reducing dough stability and decreasing the porosity during fermentation [46]. This is likely why the bread in this study had fewer air bubbles and a smaller height.

The effect of BBL substitution on the color of bread also differed between BW and 1BS-18M. The L* value (lightness) tended to decrease in both the BW and 1BS-18M bread types with an increase in BBL substitution. However, the a* value (red-green) increased in BW and decreased in 1BS-18M with an increase in the BBL-substitution rate. The b* value (blue–yellow) increased in BW with the increase in the BBL-substitution rate. No consistent trend was observed for 1BS-18M (Figure 7). The difference in the effect of BBL addition on the a* and b* values of BW and 1BS-18M bread is thought to be due to the original color of the raw materials, BW and 1BS-18M.

As shown in Figure 1, the colors of BW and 1BS-18M are very different. Compared with BW, 1BS-18M has a lower L* value and higher a* and b* values, and the bran fragments are visible to the naked eye. Therefore, it can be suggested that the L* value of the both the BW and 1BS-18M bread varieties decreased because of BBL substitution, which has a lower L* value than both BW and 1BS-18M, as well as to the baking-induced amino–carbonyl reaction [47]. The a* values of BW and 1BS-18M were 0.23 and 3.15, respectively, both of which were higher than that of BBL (−1.15). Therefore, we hypothesized that the a* values would decrease as the BBL-substitution rate increased. However, this did not happen, probably owing to the browning caused by the amino–carbonyl reaction as the baking progressed [48,49]. As the a* value of BW was close to that of BBL, the increase in the a* value due to browning was greater in the BW bread than that obtained by BBL substitution alone. In addition, although the a* value decreased in the 1BS-18M bread as the BBL-substitution rate increased, the a* value of the 1BS-18M raw material was significantly higher than that of BBL. Therefore, it can be suggested that the effect of BBL substitution is greater than that of the browning caused by the amino–carbonyl reaction, which could explain the decrease in the a* value. In addition, the b* values were 8.81 for BW and 9.61 for 1BS-18M, both of which were lower than that for BBL (23.92). Therefore, we predicted that the b* value would increase as the BBL-substitution rate increased, and while BW followed the expected trend, 1BS-18M did not follow any particular trend. As can be seen from the appearance, BW has high brightness and is close to white. In contrast, 1BS-18M is made of whole-wheat flour. Therefore, bran is mixed in 1BS-18M, and the color becomes different in some of its parts, making it darker overall (Figure 6a). The yellowness of the added ingredients is clearly reflected in the color of the bread. The b* value increases with an increase in the BBL-substitution rate. However, even when the substitution rate of BBL is increased, the 1BS-18M bread remains dark and does not become yellowish. This could be easily observed with the naked eye (Figure 6a). Hence, it can be suggested that the effect of BBL substitution on color was more likely to be seen in BW than in 1BS-18M, most likely because 1BS-18M is whole-wheat flour. Therefore, it is necessary to examine the effect of bread color by adding BBL using flour milled under the same conditions.

### 3.5. Specific Volume and Textural Properties

**Figure 8:**
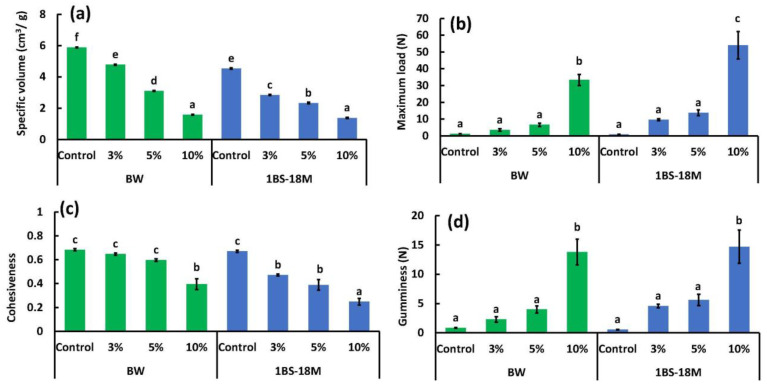
Effect of the BBL substitution rate on the (**a**) specific volume, (**b**) maximum load, (**c**) cohesiveness, and (**d**) gumminess. BW, bread wheat; 1BS-18M, 1BS-18 “Minaminokaori”; BBL, bayberry leaf. The results were analyzed using ANOVA followed by Tukey’s test for multiple comparisons. Different letters indicate significant differences at *p* < 0.05. Data are expressed as the mean ± SE. Specific volume, *n* = 3; maximum load, cohesiveness, and gumminess, *n* = 10.

Figure 8 shows the data for the specific volume (a), maximum hardness load (b), cohesiveness (c), and gumminess (d). The specific volumes of both BW and 1BS-18M significantly decreased with an increase in BBL substitution. In BW, the specific volumes of the control and the BW bread with 3%, 5%, and 10% BBL substitution were 5.9, 4.8, 3.1, and 1.6 cm^3^/g, respectively. The corresponding values for 1BS-18M were 4.5, 2.8, 2.3, and 1.4 cm^3^/g, respectively. The texture of bread is closely related to its specific volume. The higher the specific volume, the softer the texture of the bread will generally be, and the more desirable the texture of the bread will be. A previous study used gluten-free bread formulations to determine the specific volume as an indicator of quality improvement [50]. In our study, specific volume decreased in a concentration-dependent manner with an increase in the BBL-substitution rate. In addition, a comparison of breads with the same BBL-substitution rate showed that the specific volume of 1BS-18M was lower than that of BW. The air-bubble structure of bread has a significant effect on its texture and is therefore an extremely important factor that determines the bread quality. The fact that the specific volume decreased as the BBL-substitution rate increased indicates that the increase in the BBL-substitution rate prevented the bread from maintaining its air-bubble structure, which decreased the bread volume.

The most important factor in the generation and retention of bubbles is the formation of gluten. Gluten formation requires gliadin, which gives the dough flexibility, and glutenin, which gives it elasticity [51]. As mentioned above, an increase in the BBL-substitution rate reduces the amount of protein in the bread. It also reduces the amount of gliadin present in the bread (Figure 4). As a result, the amount of gluten decreases, making it difficult to form a gluten network. In addition to the effects of reduced protein mass, several other factors interfere with the formation of a gluten network as well, such as PAs and dietary fiber. Because BBL contains 6756 mg CTN eq./100 g DW of PAs (Figure 1) and 53.2 g/100 g DW of dietary fiber (Table 1), both of which are very high values, it is possible that these components are responsible for the decrease in the protein content of bread because of the increase in the BBL-replacement rate.

As mentioned in Section 3.2.2, PAs have phenolic hydroxyl groups and hydrophobic regions. They can form complexes with the carbonyl groups of proteins and hydrophobic amino acids [12], which may interfere with the formation of the gluten network. Dietary fiber induces the aggregation of gluten proteins into hydrogen-bonded beta-sheets, which may involve other beta-sheets, anti-parallel beta-sheets, beta-turns, and/or alpha-helices. This aggregation is believed to be a result of competition for water molecules between gluten proteins and dietary fiber polysaccharides, which reportedly leads to the partial dehydration of the gluten matrix [52,53,54]. Hence, it can be hypothesized that the decrease in specific volume due to the increase in the BBL-substitution rate was a result of the combination of the following factors: a decrease in protein content due to BBL substitution; the inhibition of gluten network formation, which is a protein network formed by the binding of PA and protein; and the partial dehydration of the gluten matrix by dietary fiber. A decrease in specific volume, or poor bulking, has a significant impact on textural properties. There was no significant difference in the maximum load between the control and BBL3% and 5% substitution breads. However, the BBL10% substitution bread (BW: 33.38 ± 3.29 N; 1BS-18M: 54.03 ± 8.19 N) had a significantly higher maximum load than that of the other bread types (*p* < 0.05) (Figure 8b).

Bread with a high maximum load is harder [55,56]. Cohesiveness in bread indicates how well a bread type can maintain its structure when compressed or bitten. Bread with a high cohesiveness is perceived as having a good texture and to be easier to chew and swallow [57]. In this study, the use of BW did not cause any significant difference in the cohesiveness of the control or the BBL3%, and BBL5% substitution breads. Only the BBL10% substitution bread had a significantly lower cohesiveness (Figure 8c). In the case of 1BS-18M, the control had a cohesiveness of 0.67, while the BBL3% and 5% breads had cohesiveness values of 0.47 and 0.39, respectively, and the BBL10% group had a significantly lower value (0.25) (Figure 8c). Thus, cohesiveness was significantly lower for the BW and 1BS-18M 10% BBL-substituted breads, revealing a poor texture. Gumminess indicates firmness at the second bite. The BW and 1BS-18M breads with 10% BBL substitution showed significantly higher values than the other treatments (*p* < 0.05) (Figure 8d). The BW and 1BS-18M 10% BBL-substituted breads showed significantly higher gumminess values of 13.79 and 14.71, respectively, than other treatments, indicating significantly higher firmness at the second bite. From the above, the results of the texture characteristic analysis showed that 10% BBL substitution in both BW and 1BS-18M resulted in bread with a poor texture that significantly impaired the quality of the bread, and that from the perspective of texture, BBL substitution should be limited to 5%.

## 4. Conclusions

We aimed to obtain a hypoallergenic wheat product using BBL, a discarded product from pruning. By replacing a portion of BW and low-allergen wheat 1BS-18M (3%, 5%, and 10% by weight) with BBL, it was found that the reduction of allergens in bread was possible with an increasing replacement rate, as shown by multiple evaluation systems such as SDS-PAGE, WB, and ELISA. In particular, it was found that the allergen content of bread could be reduced to 9–29% compared with the control when BBL was substituted at a rate of 10% (Figure 4). In addition, it was found that when the substitution rate of BBL was increased, the color of both the BW and 1BS-18M breads became darker, and their TPC and antioxidative properties (DPPH value, H-ORAC value) increased significantly. In particular, when BBL was substituted at 10%, the TPC, DPPH value, and H-ORAC value increased 4.9 (1BS-18M bread)–8.6 (BW bread) times, 24.6 (BW bread)–27.2 (1BS-18M bread) times, and 43.5 (BW bread)–46.1(1BS-18M bread) times, respectively, compared with the control. Although the values for the BBL-5% substituted bread did not reach those of the BBL-10% substituted bread, the TPC, DPPH value, and H-ORAC value still increased 2.8 (1BS-18M bread)–4.3 (BW bread) times, 9.7 (BW bread)–12.0 (1BS-18M bread) times, and 15.1 (BW bread)–26.0 (1BS-18M bread) times, respectively, compared with the control. The BBL-3% substituted bread also showed increases in the TPC, DPPH value, and H-ORAC value of 2.0 (1BS-18M bread)–2.9 (BW bread) times, 4.1 (BW bread)–5.7 (1BS-18M bread) times, and 4.3 (BW bread)–19.7 (1BS-18M bread) times, respectively, compared with the control. As can be seen, BBL-substituted bread improves the TPC and antioxidative properties. As mentioned above, improving the TPC and antioxidative properties may be a solution for controlling chronic diseases such as cardiovascular complications, hypertension, diabetes, and various cancers. However, we found that BBL-10% substituted bread significantly reduced the height, specific volume, and texture of the bread. Therefore, we believe that BBL-5% substituted bread is more suitable when considering the overall balance between low allergenicity, improved antioxidative properties, and reduced quality. In this experiment, a commercially available kit was used to detect gliadin and multiple wheat-protein components. Ideally, it would be preferable to use individual or pooled sera from wheat allergy patients and to evaluate them using the secondary detection of anti-IgE, but this is a subject for future research. Furthermore, there are also a number of issues that need to be addressed in this research. These include the fact that 1BS-18M is made from whole-wheat flour, the fact that a sensory test has not yet been conducted to assess the taste, that immunodetection analysis has not yet been extended to the final bread samples after static in vitro digestion, and the fact that the low allergen effect of the product has not yet been verified through human consumption. The low allergenicity of wheat flour foods achieved through the addition of PAs that we are aiming for cannot be achieved unless the allergenic protein–PA complex can be retained through the digestive process and heat treatment in the cooking process. In the future, we plan to conduct research on the effects of digestive enzymes, pH, and heat treatment on the allergenic protein–PA complex. Food safety is an essential element of a healthy life. The results of this study suggest that it is possible to obtain low-allergenic wheat products using inexpensive and easily available BBLs, which may help make these products affordable. We hope to conduct further research and to establish production technologies for hypoallergenic and safe wheat products.

## Figures and Tables

**Table 1:** General composition and dietary fiber values of the raw materials.

		BBL	BW	1BS-18M
Energy	kcal/100 g	276	346	333
Protein	g/100 g	9.5	13.1	12.8
Fat	g/100 g	3.9	1.4	2.0
Carbohydrate	g/100 g	77.3	71.5	69.4
Dietary fiber	g/100 g	53.2	2.7	7.1
Ash	g/100 g	2.9	0.2	1.2

BBL, bayberry leaf; BW, bread wheat; 1BS-18M, 1BS-18 “Minamino Kaori”.

## Data Availability

The original contributions presented in the study are included in the article, further inquiries can be directed to the corresponding author.
